# Supplementary figures and images for: Licochalcone A Inhibits Prostaglandin E2 by Targeting the MAPK Pathway in LPS Activated Primary Microglia
Source: Molecules. 2023 Feb 17;28(4):1927. doi: 10.3390/molecules28041927 (PMC9965579; doi:10.3390/molecules28041927)

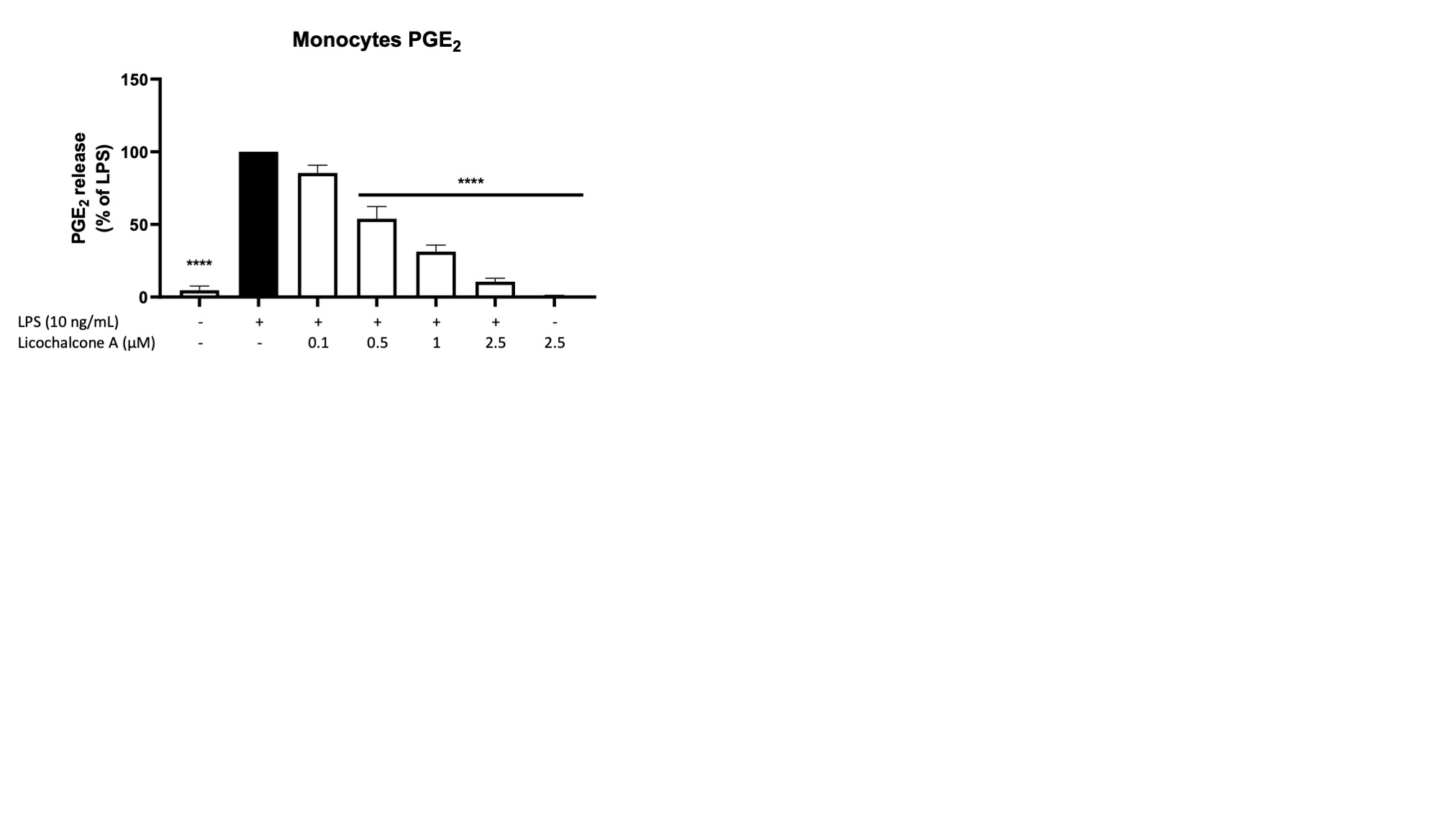

Supplement: Supplementary file 1 [file molecules-28-01927-s001.zip › molecules-2166444-supplementary.jpeg]
